# Xq28 (MECP2) microdeletions are common in mutation-negative females with Rett syndrome and cause mild subtypes of the disease

**DOI:** 10.1186/1755-8166-6-53

**Published:** 2013-11-27

**Authors:** Ivan Y Iourov, Svetlana G Vorsanova, Victoria Y Voinova, Oxana S Kurinnaia, Maria A Zelenova, Irina A Demidova, Yuri B Yurov

**Affiliations:** 1 Mental Health Research Center, Russian Academy of Medical Sciences, Moscow 117152, Russia; 2 Institute of Pediatrics and Children Surgery, Ministry of Health of the Russian Federation, Moscow 125412, Russia; 3 Department of Medical Genetics, Russian Medical Academy of Postgraduate Education, Moscow 123995, Russia; 4 Moscow City University of Psychology and Education, Moscow 127051, Russia

**Keywords:** Array CGH, Autistic spectrum disorder, Chromosome X, Female, *MECP2*, Rett syndrome, Xq28 microdeletion

## Abstract

**Background:**

Rett syndrome (RTT) is an X-linked neurodevelopmental disease affecting predominantly females caused by *MECP2* mutations. Although RTT is classically considered a monogenic disease, a stable proportion of patients, who do not exhibit *MECP2* sequence variations, does exist. Here, we have attempted at uncovering genetic causes underlying the disorder in mutation-negative cases by whole genome analysis using array comparative genomic hybridization (CGH) and a bioinformatic approach.

**Results:**

Using BAC and oligonucleotide array CGH, 39 patients from RTT Russian cohort (in total, 354 RTT patients), who did not bear intragenic *MECP2* mutations, were studied. Among the individuals studied, 12 patients were those with classic RTT and 27 were those with atypical RTT. We have detected five 99.4 kb deletions in chromosome Xq28 affecting *MECP2* associated with mild manifestations of classic RTT and five deletions encompassing *MECP2* spanning 502.428 kb (three cases), 539.545 kb (one case) and 877.444 kb (one case) associated with mild atypical RTT. A case has demonstrated somatic mosaicism. Regardless of RTT type and deletion size, all the cases exhibited mild phenotypes.

**Conclusions:**

Our data indicate for the first time that no fewer than 25% of RTT cases without detectable *MECP2* mutations are caused by Xq28 microdeletions. Furthermore, Xq28 (*MECP2*) deletions are likely to cause mild subtypes of the disease, which can manifest as both classical and atypical RTT.

## Background

Rett syndrome (RTT // MIM 312750) is an X-linked neurodevelopmental disorder caused by *MECP2* mutations that affects almost exclusively girls. Clinically, the disease presents with developmental regression accompanied by the loss of hand skills, mobility and speech. In addition, RTT is phenotypically characterized by stereotypic hand movements, respiratory abnormalities, scoliosis, growth deficits, hypotonia, microcephaly and seizures. Intragenic *MECP2* mutations are the main cause of RTT. However, there does exist a proportion of RTT females (5-10%) without detectable *MECP2* mutations [1-6]. To date, genetic causes in these RTT cases remain largely unknown.

Recently, it has been shown that Xq28 microdeletions can affect *MECP2* leading to RTT-like phenotype [7,8]. Since these submicroscopic genome variations were commonly detected in children with presumably idiopathic intellectual disability, autism, epilepsy and/or congenital anomalies [7], it is probable that submicroscopic Xq28 deletions are not rare and can be associated with RTT. In this context, one can suggest Xq28 deletions spanning the *MECP2* gene to be a potential cause of the disease in affected females without mutations detectable by Sanger sequencing. Surprisingly, to the best our knowledge, there was no systematic whole genome analysis of *MEPC2*-mutation negative RTT patients. In the available literature, we have only found studies describing whole genome analysis of RTT females by array comparative genomic hybridization (CGH), which was performed for testing whether copy number variants (CNVs) are able to modulate the phenotype in mutation-positive RTT cases [9,10]. Thus, we decided to share our data on the evaluation of *MEPC2*-mutation negative females from Russian RTT cohort addressed by BAC and oligonucleotide array CGH with bioinformatic analysis.

## Results

**Figure 1:**
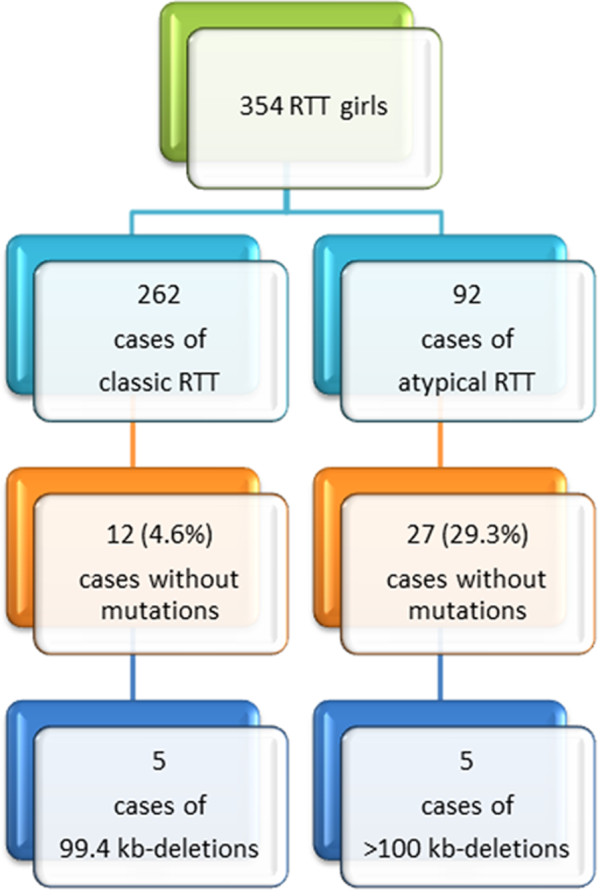
Flow chart illustrating the diagnostic workup for genetic evaluation in Russian RTT cohort.

In the present study, we have selected *MECP2-*mutation-negative patients from the Russian RTT cohort (354 RTT girls). The cohort includes 262 classic and 92 atypical RTT females according to revised diagnostic criteria [11], who have been previously found to bear a *MECP2* mutation in 95.4% and 70.7% of cases, respectively [12-15]. The remaining RTT girls were classified as follows: classic RTT — 12 cases out of 262 patients (4.6%) and atypical RTT — 27 cases, among them 17 girls with “forme fruste”; 6 with a late regression; 4 girls with early-onset seizures. All the selected patients (n = 39) have been evaluated by BAC and oligonucleotide array CGH (Human BAC Array-System, Perkin Elmer and NimbleGen 135 K whole genome tiling array) using a specific bioinformatic protocol for data analysis. Five classic RTT cases and five atypical RTT cases were found to be associated with Xq28 deletions (Figure 1). The occurrence of Xq28 deletions in RTT females without *MECP2* mutations detectable by Sanger sequencing was estimated at about 26%. These cases were all found to exhibit RTT-specific epigenetic phenomena (unusual replication pattern or type C undetectable in general population) observed at cytogenetic (cytological) level.

**Figure 2:**
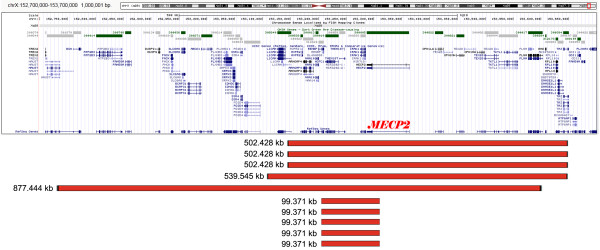
**Schematic overview of detected Xq28 deletions depicted using UCSC Genome Browser (Human Feb. 2009 (GRCh37/hg19) Assembly) (see also Table**1**for details).**

Firstly, the deletions were detected in girls with atypical RTT by BAC array CGH. Oligonucleotide array CGH was then used to confirm the deletions and to narrow the breakpoints given according to hg19 assembly (Feb. 2009 Genome Reference Consortium GRCh37). Among them, three patients exhibited exactly the same (recurrent) deletions encompassing genomic loci in Xq28: arr Xq28(153,145,800-153,648,227)×1 (Additional file 1: Figure S1). The size of these three deletions was estimated as 502,428 bp. Another RTT patient has demonstrated an Xq28 deletion with the same distal breakpoint (arr Xq28(153,108,683-153,648,227)×1), the size of which is 539,545 bp (Additional file 2: Figure S2). The largest Xq28 deletion detected in this study spans 877,444 bp (arr Xq28(152,731,931-153,609,374)×1) and is featured by an unexpectedly mild RTT phenotype (Additional file 3: Figure S3). All the deletions detected in atypical RTT cases were found to encompass the *MECP2* gene (Figure 2). Deletions were confirmed by fluorescence *in situ* hybridization (FISH). Molecular data (size and breakpoint locations) and clinical parameters (additional phenotypic features) of these deletions are summarized in Table 1.

**Table 1:** **Clinical and molecular overview of Xq28 microdeletions detected in *MECP2-*** **mutaiton-negative RTT females**

**Age (months)**	**Additional clinical features**	**Size (kb)**	Breakpoints*	
			**Proximal**	**Distal**
Atypical RTT				
118	Multiple hematomas, teeth anomalies	502.428	153,145,800	153,648,227
132	Prenatal hypotrophy, facial dysmorphisms	502.428	153,145,800	153,648,227
58	Prenatal hypotrophy, facial dysmorphisms, clinodactyly, dentinogenesis imperfecta, cerebellar vermis hypoplasia, epidural cystic changes in the thoracic spine	502.428	153,145,800	153,648,227
48	Prenatal hypotrophy, facial dysmorphisms, verrucous patches on the trunk, patent foramen ovale	539.545	153,108,683	153,648,227
22	Facial dysmorphisms	877.444	152,731,931	153,609,374
Classic RTT				
204	Prenatal hypotrophy, facial dysmorphisms	99.371	153,213,483	153,312,854
74	Hydronephrosis, polycystic kidney disease	99.371	153,213,483	153,312,854
49	Prenatal hypotrophy, facial dysmorphisms	99.371	153,213,483	153,312,854
101**	—	99.371	153,213,483	153,312,854
98	Prenatal hypotrophy	99.371	153,213,483	153,312,854

* — according to assembly: hg19 Feb. 2009 Genome Reference Consortium GRCh37;

** — somatic mosaicism and discrepancy between array CGH and FISH data.

**Figure 3:**
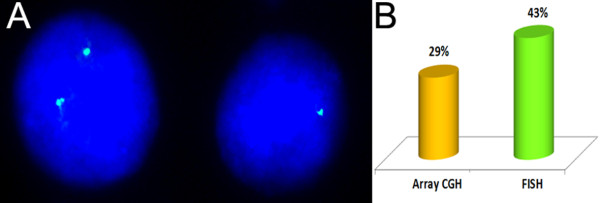
**FISH demonstrating mosaic *MECP2 *deletion. (A)** interphase FISH: two signals correspond to two *MECP2* copies in a nucleus without deletion and a single signal is observed in a nucleus lacking one *MECP2* copy; **(B)** percentages of abnormal cells detected by array CGH and FISH.

Secondly, oligonucleotide array CGH has indentified another five deletions in nearly classic RTT patients. All these deletions have the same breakpoints (same size) (Table 1, Additional file 4: Figure S4): about 99.4 kb (arr Xq28(153,213,483-153,312,854) ×1). One case was found to be associated with mosaic deletion (Table 1), which was confirmed by FISH through studying 100 metaphase plates and 1000 interphase nuclei (unfortunately, other tissues were not available for analysis due to parents’ lack of further cooperation). According to molecular analysis, two *MECP2* exons were affected (Figure 2, Additional file 4: Figure S4). However, taking into account the probe distribution on the NimbleGen 135 K whole genome tiling array, complete *MECP2* deletion on one chromosome X homologue cannot be excluded. It is intriguing to note that further molecular cytogenetic confirmation analysis demonstrated a discrepancy between array CGH and FISH, which mainly concerned the mosaic case hallmarked by an apparent difference between proportions of cells affected by *MECP2* deletion (Figure 3). Apart from Xq28 deletions, other CNVs were also detected. These were losses within 3p13, 3q27.1, 11p13, 15q11.2, Xp22.13 and gains within 1q21, 11p14.3, 15q14, 22q11.21. We recognize that their intrinsic pathogenic value can be appreciable and requires to be addressed by further bioinformatics and molecular analyses (more detailed data will be presented elsewhere).

An attempt at correlation between genotype and phenotype in reported cases and cases with *MECP2* mutations has shown that Xq28 (*MECP2*) microdeletions are likely to cause specific subtypes of RTT, which are clinically milder than the phenotype resulted from intragenic *MECP2* sequence variations. Deletions were featured by late regression age, intact ability to walk, mild dyspraxia of hand movements, and microcephaly absence. Although some cases fulfil both canonical and more recent diagnostic criteria for classic RTT [11,16], we did observe that almost all the RTT symptoms (addressed by a scale developed specifically for the evaluation of RTT symptoms severity [8,14,15]) in cases of Xq28 deletions are comparably milder than those caused by intragenic *MECP2* mutations. Additional clinical signs featuring this RTT subtype are low birth weight in ~2/3 of cases, malformations (vascular dorsal skin hemangiomatosis, verrucous patches resembling incontinentia pigmenti phenotype, cerebellar vermis hypoplasia, polycystic kidney disease, patent foramen ovale) and facial dysmorphisms.

## Discussion

RTT is a common monogenic cause of neurodevelopmental abnormalities in females [1-6]. Although it has been repeatedly noted that the phenotype of affected girls depends on the presence or absence of *MECP2* mutation, the latter have not been ever considered as an exclusive criterion for RTT [11,16,17]. Apparently, non-striking phenotypic differences between a significant proportion of mutation-positive and mutation-negative cases [17] indicate that the same genetic defect causes the disease in mutation-negative cases. Currently, there have been reported several genomic abnormalities (i.e. 14q12 microdeletions) associated with RTT [5-7,18,19]. However, these genomic rearrangements are unlikely to cover all the mutation-negative RTT cases. Here, we report on the commonest cause of RTT in cases without detectable *MECP2* mutations. To our knowledge, this is the first systematic report describing Xq28 genomic abnormities (Xq28 deletions affecting *MECP2*) in RTT.

Large intragenic *MECP2* deletions have been consistently reported in the available literature [20-24]. Moreover, a RTT case was associated with a deletion detected by FISH [20]. Nevertheless, the existence of Xq28 deletions causing RTT has long remained a matter of conjecture. It seems that the high mutation detection rate and clinical heterogeneity in mutation-negative cases has resulted in the lack of studies dedicated to whole genome analysis among RTT females without detectable *MECP2* mutations. On the other hand, *MECP2* loss modulates RTT phenotypes in mice [25,26] suggesting that similar genomic abnormality might cause RTT in humans. Xq28 (*MECP2*) deletions found in RTT girls has confirmed this expectation. Furthermore, studying functional consequences of *MECP2* mutations [27-29] evidences that *MECP2* loss has functional implications in females.

As detected by array CGH and FISH, one deletion causing classic RTT was mosaic. Somatic mosaicism for a structural chromosome abnormality or CNVs is common in genomic disorders or single-gene disease [30,31]. It is also detected in cohorts of individuals with autistic spectrum disorders (in its widest sense) including girls suffering from RTT [12,18,32-35]. This makes it attractive to analyze molecular and clinical aspects of Xq28 (*MECP2*) deletions in the light of increasing interest in biomedical studies of autism, especially considering the positive experience in modelling neurodevelopmental abnormalities according to data on RTT pathogenesis [36,37]. To explain differences between cell proportions uncovered by array CGH and FISH (Figure 3), one can compare molecular cytogenetic techniques in context of detecting somatic mosaicism [38-40]. In this instance, we have concluded that FISH results are more accurate. Similarly, FISH questioned in some detail the size of the recurrent deletion causing classic RTT. Since oligonucleotide probes cover a part of *MECP2* sequence whereas the deletion was detectable by FISH with a probe for *MECP2*, we have speculated that genomic loss within Xq28 is a bit larger than shown by the array CGH. Likewise, sequence variations specifically generating Xq28 subchromosomal rearrangements are co-localized with the breakpoints outside of *MECP2* loci [41,42]. So far, it appears to be also valid for reported deletions. To determine the intrinsic nature and causes of Xq28 (*MECP2*) deletions leading to classic RTT, further studies are indisputably required.

The specific replication patterns in RTT or type C (observed in about 90% of affected children in contrast to unaffected females [12,43]) have been detected in females with Xq28 microdeletions. The type C replication pattern represents a disturbance in the sequence of replication in an inactive chromosome X apparently caused by *MECP2* mutations [12,15]. These data allowed speculations that RTT in mutation-negative females is likely to be associated with genetic defects affecting the *MECP2* gene [15]. Array CGH analysis of RTT girls, highlighting Xq28 (*MECP2*) deletion as a new cause of the disease, confirms this assumption.

Although RTT phenotype is characterized by recognizable patterns of malformation and distinct neurodevelopmental abnormalities, there does exist a clinical variability among females suffering from this severe disorder [3-6,11,14,16,17]. Xq28 deletions causing atypical RTT have shown to exhibit additional phenotypic features (Table 1). This can be easily explained, because all deletions have spanned significantly larger regions than the *MECP2* locus, involving other Xq28 genes, as well (Figure 2). Conversely, Xq28 losses (*MECP2* plus some additional genes) should naturally lead to the presence of phenotypic manifestations usually unseen in RTT. Interestingly, RTT females with large Xq28 deletions have demonstrated less severe disease manifestations as compared to their counterparts with intragenic *MECP2* mutations of known functional consequences. This is likely to result from X chromosome inactivation skewing probably arisen from selective disadvantages of cells with an active deleted chromosome X. In the same way, *MECP2* deletions causing classic RTT are likely to lead to less severe RTT manifestations through the skewed X chromosome inactivation patterns. Thus, epigenetic phenotype modulators determine the outcome of subchromosomal deletions involving *MECP2*. This has led us to the conclusion that, regardless of specific phenotypic appearance, the Xq28 deletion phenotype is not different enough from RTT due to intragenic *MECP2* mutations to define it as an independent clinical entity or a microdeletion syndrome. Summarizing the clinical data on girls found to demonstrate Xq28 (*MECP2*) microdeletions, we have concluded that these genomic rearrangements cause at least two distinct RTT subtypes. The first subtype is caused by deletions spanning from 0.5 to 1 Mb and is characterized by less severe RTT manifestations as well as additional clinical signs. The second subtype is caused by deletions spanning about 100 kb leading to a loss of *MECP2 per se* and is simply a less severe classic RTT. Finally, both types can be arbitrarily designated as microdeletion RTT subtype.

To this end, it is to mention that submicroscopic genomic variations and CNVs are likely to be among the commonest causes of congenital malformations, idiopathic intellectual disability, autism, epilepsy, neuropsychiatric disorders [18,36,44]. Seemingly, these genome variations are likely to be important elements of pathogenetic cascades in complex disease mediating genetic-environmental interactions [45]. The present study evidences that submicroscopic deletions or CNVs cause single-gene disorders in an appreciable proportion of cases.

## Conclusions

Using two array CGH platforms (BAC and oligonucleotide array CGH) and FISH, the existence of Xq28 deletions causing RTT was shown. To date, such genomic deletions were not actually recognized as a cause of RTT, a disease considered to be almost exclusively monogenic. We show that Xq28 (*MECP2*) deletions are common in RTT girls without detectable *MECP2* sequence variations by Sanger sequencing affecting no fewer than 25% of mutation-negative females. Therefore, the efficiency of molecular diagnosis can be significantly increased through applying whole genome scan to mutation-negative RTT cases. Our data evidence that there exist at least two types of Xq28 microdeletions affecting *MECP2*: small deletions spanning about 100 kb and larger deletions spanning >100 kb (0.5-1 Mb). The former is likely to cause mild classic RTT, whereas the latter seems to result in mild atypical RTT forms. Finally, we conclude that Xq28 (*MECP2*) deletions are common in mutation-negative RTT girls and cause mild subtypes of the disease.

## Methods

### Patients

Thirty nine *MECP2*-mutation-negative females were recruited for molecular cytogenetic analysis according to molecular genetic data from the Russian RTT cohort (354 patients). All the girls fulfill clinical criteria for RTT either classic or atypical form. The information about Russian RTT cohort was provided previously [8,12,14,15] and is partially given in Figure 1. The DNA samples studied were isolated from peripheral blood leukocytes following standard techniques. Written informed consent was obtained from the patients’ parents. The research was approved by the ethical committee at the Mental Health Research Center (Russian Academy of Medical Sciences) and by Russian Rett Syndrome Association.

### Sequencing

The performance and results (partially) of Sanger sequencing was previously described [14,15,46]. The lack of a sequence variation in *MECP2* known to be associated with RTT or to have a functional consequence was a criterion for entry into the study.

### Array CGH

BAC-array CGH was performed using customized Constitutional Chip®4.0 (Human BAC Array-System, Perkin Elmer, USA) as described earlier [7,47]. The resolution of the BAC-array has been estimated as 0.3 Mb for scanning chromosome X.

Oligonucleotide array CGH was performed using NimbleGen 135 K whole genome tiling array (described in parts by Duker et al. [48]). The calculated functional resolution was estimated 10–20 kb (95% confidence). Sample and reference DNA was labeled using Cy3-dUTP and Cy5-dUTP, respectively, and hybridized according to the manufacturer’s protocols (NimbleGen Arrays User’s Guide CGH and CGH/LOH Arrays v9.1, Roche NimbleGen, Madison, WI, USA). Scanning and image acquisition has been processed in the same way as for BAC-Perkin Elmer Array [7,47].

### FISH

FISH (probe labeling, hybridization and detection) was performed according to previously described protocols [12,13,35,49]. The DNA probe was a YAC (yeast artificial chromosome) containing almost exactly *MECP2* sequence and was kindly provided by Dr. Maurizio D’Esposito (Naples, Italy). The probe (localization and DNA sequence) was described previously [50].

### Cytogenetic/cytological analysis of epigenetic phenomena

Unusual replication pattern or type C (detectable in nearly 90% of RTT children and unobserved in females without RTT [12,43,49]) i.e. disturbances in the replication sequence of an inactive chromosome X, was assessed by replication staining of metaphase chromosomes obtained from cultivated peripheral blood lymphocytes in the presence of 5-bromo-20-deoxyuridine as described in detail earlier [12,43]. The presence of type C was evaluated by analyzing 50–100 metaphase plates.

### Data analysis (bioinformatics)

The raw array CGH data (log 2 intensity ratios) were processed for CNV detection as proposed earlier [51,52]. The protocol was modified to achieve comprehensive data on CNVs according to intensity ratios values for 4 oligonucleotide and 2 interchangeable BAC probes. Using different threshold schemes and background correction, the intensity ratios corresponding to CNVs spanning *MECP2* were established empirically. Localization of probes in the oligonucleotide array CGH assay corresponding to *MECP2* sequence was as follows: 153,299,881; 153,306,195; 153,308,602; 153,312,854. In BAC array CGH assay, there were 3 BAC probes for the X chromosome sequence encompassing the *MECP2* gene. It is to note, that deletions have spanned larger regions than those covered by the probes strictly corresponding to *MECP2* loci. The established threshold allowed the detection of non-mosaic and mosaic CNVs through the comparison of mean values of chromosome-specific intensity ratios and mean values of a locus of interest in Xq28.

## Abbreviations

CNVs: Copy number variations; CGH: Comparative genomic hybridization; FISH: Fluorescence *in situ* hybridization; MECP2: Gene encoding methyl-CpG binding protein 2; RTT: Rett syndrome; YAC: Yeast artificial chromosome.

## Competing interests

The authors declare that they have no competing interests.

## Authors’ contributions

IYY, SGV and YBY conceived the research, designed the study, and wrote the manuscript; IYY, SGV, VYV and YBY conceived the project and obtained the funding; VYV and SGV referred the patients for the study; IYI, OSK, MAZ and IAD performed the experiments and participated in the diagnostic service. All authors have read and approved the final manuscript.

## Authors’ information

Ivan Y Iourov and Svetlana G Vorsanova are joint first authors.

## Supplementary Material

Additional file 1: Figure S1 The deleted Xq28 region spanning 502.428 kb displayed using UCSC Genome Browser on Human Feb. 2009 (GRCh37/hg19) Assembly (http://genome-euro.ucsc.edu/index.html). Click here for file

Additional file 2: Figure S2 The deleted Xq28 region spanning 539.545 kb displayed using UCSC Genome Browser on Human Feb. 2009 (GRCh37/hg19) Assembly (http://genome-euro.ucsc.edu/index.html). Click here for file

Additional file 3: Figure S3 The deleted Xq28 region spanning 877.444 kb displayed using UCSC Genome Browser on Human Feb. 2009 (GRCh37/hg19) Assembly (http://genome-euro.ucsc.edu/index.html). Click here for file

Additional file 4: Figure S4 The deleted Xq28 region spanning 99.371 kb displayed using UCSC Genome Browser on Human Feb. 2009 (GRCh37/hg19) Assembly (http://genome-euro.ucsc.edu/index.html). Click here for file
