# Supplementary figures and images for: Xq28 (MECP2) microdeletions are common in mutation-negative females with Rett syndrome and cause mild subtypes of the disease
Source: Mol Cytogenet. 2013 Nov 27;6:53. doi: 10.1186/1755-8166-6-53 (PMC4176196; doi:10.1186/1755-8166-6-53)

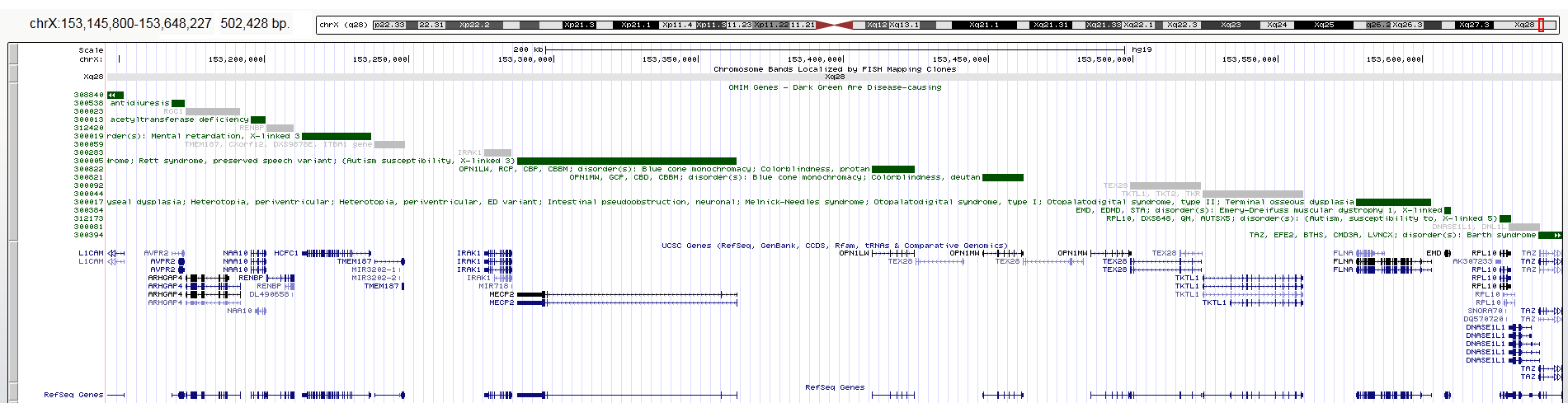

Supplement: Additional file 1: Figure S1 — The deleted Xq28 region spanning 502.428 kb displayed using UCSC Genome Browser on Human Feb. 2009 (GRCh37/hg19) Assembly (http://genome-euro.ucsc.edu/index.html). [file 1755-8166-6-53-S1.tiff]

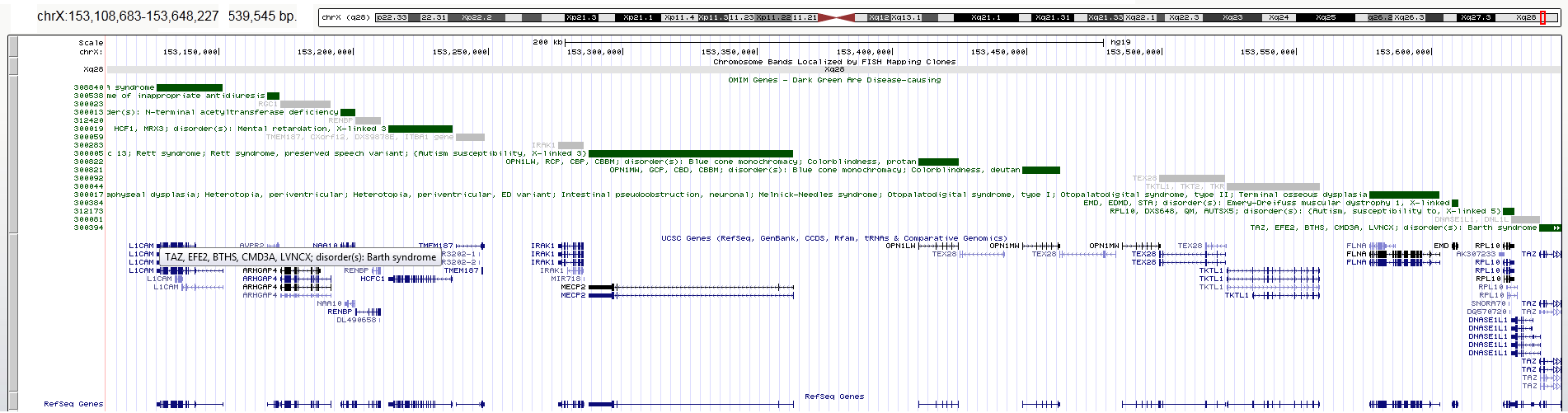

Supplement: Additional file 2: Figure S2 — The deleted Xq28 region spanning 539.545 kb displayed using UCSC Genome Browser on Human Feb. 2009 (GRCh37/hg19) Assembly (http://genome-euro.ucsc.edu/index.html). [file 1755-8166-6-53-S2.tiff]

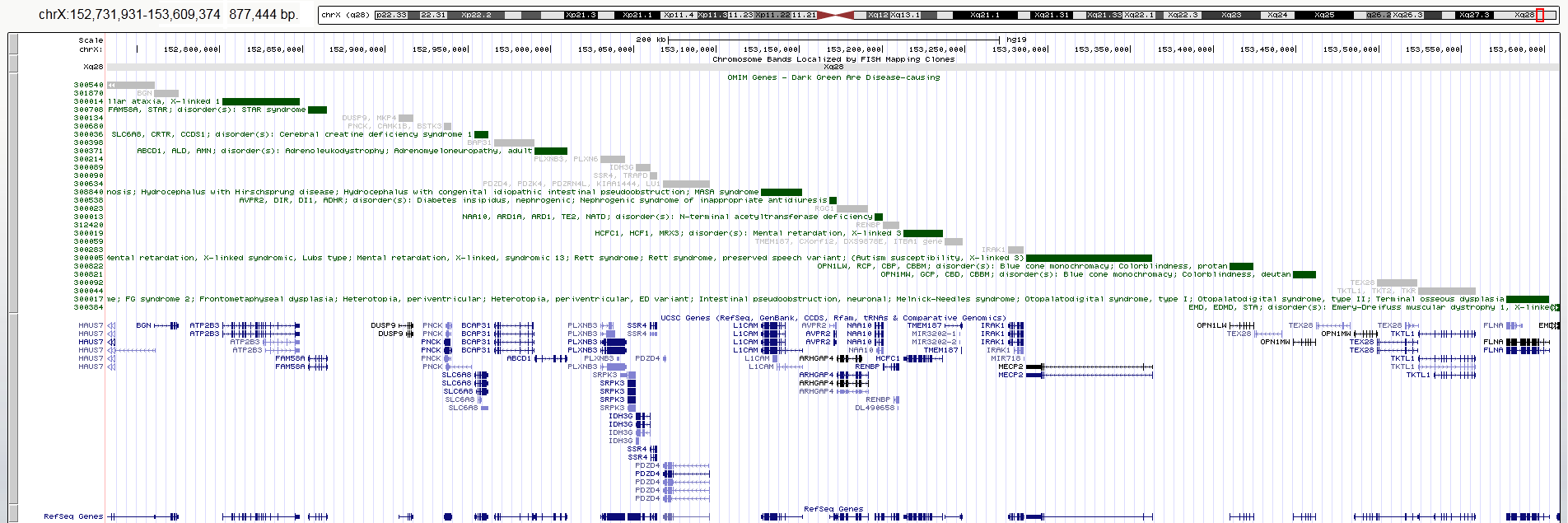

Supplement: Additional file 3: Figure S3 — The deleted Xq28 region spanning 877.444 kb displayed using UCSC Genome Browser on Human Feb. 2009 (GRCh37/hg19) Assembly (http://genome-euro.ucsc.edu/index.html). [file 1755-8166-6-53-S3.tiff]

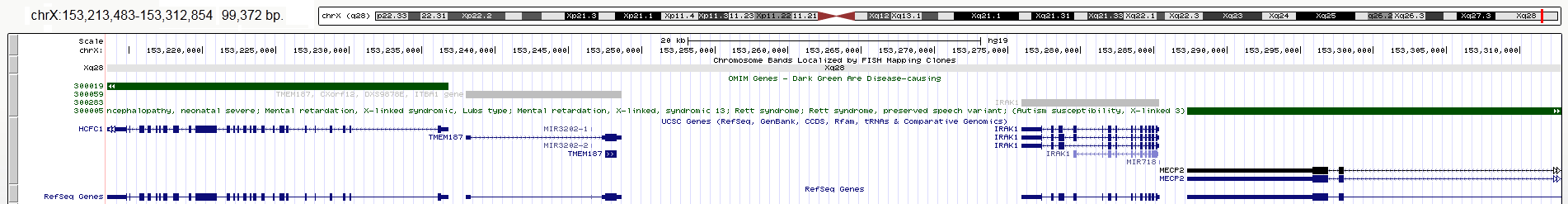

Supplement: Additional file 4: Figure S4 — The deleted Xq28 region spanning 99.371 kb displayed using UCSC Genome Browser on Human Feb. 2009 (GRCh37/hg19) Assembly (http://genome-euro.ucsc.edu/index.html). [file 1755-8166-6-53-S4.tiff]
